# The Dynamics of the Antimicrobial Resistance Mobilome of *Salmonella enterica* and Related Enteric Bacteria

**DOI:** 10.3389/fmicb.2022.859854

**Published:** 2022-03-31

**Authors:** Suad Algarni, Steven C. Ricke, Steven L. Foley, Jing Han

**Affiliations:** ^1^Division of Microbiology, FDA National Center for Toxicological Research, Jefferson, AR, United States; ^2^Cellular and Molecular Biology Graduate Program, University of Arkansas, Fayetteville, AR, United States; ^3^Meat Science and Animal Biologics Discovery Program, Department of Animal and Dairy Sciences, University of Wisconsin, Madison, WI, United States

**Keywords:** mobilome, conjugation, mobile genetic elements, *Salmonella enterica*, horizontal gene transfer

## Abstract

The foodborne pathogen *Salmonella enterica* is considered a global public health risk. *Salmonella enterica* isolates can develop resistance to several antimicrobial drugs due to the rapid spread of antimicrobial resistance (AMR) genes, thus increasing the impact on hospitalization and treatment costs, as well as the healthcare system. Mobile genetic elements (MGEs) play key roles in the dissemination of AMR genes in *S. enterica* isolates. Multiple phenotypic and molecular techniques have been utilized to better understand the biology and epidemiology of plasmids including DNA sequence analyses, whole genome sequencing (WGS), incompatibility typing, and conjugation studies of plasmids from *S. enterica* and related species. Focusing on the dynamics of AMR genes is critical for identification and verification of emerging multidrug resistance. The aim of this review is to highlight the updated knowledge of AMR genes in the mobilome of *Salmonella* and related enteric bacteria. The mobilome is a term defined as all MGEs, including plasmids, transposons, insertion sequences (ISs), gene cassettes, integrons, and resistance islands, that contribute to the potential spread of genes in an organism, including *S. enterica* isolates and related species, which are the focus of this review.

## Introduction to *Salmonella enterica*

*Salmonella enterica* is a facultative intracellular microorganism that is one of the more important etiological agents of foodborne illnesses and has a significant impact on global human health. *Salmonella* can be classified, based on the acquisition of *Salmonella* pathogenicity islands (SPIs), into two species: *S. enterica* and *Salmonella bongori* (Majowicz et al., 2010; Moreno Switt et al., 2012). Annually, more than 90 million human cases of *Salmonella* gastroenteritis are estimated globally (Mather et al., 2013). *Salmonella* infections cost an estimated $3 billion per year in both the European Union and in the United States (U.S.). There are around 1.03 million infections and 400 deaths annually in the United States due to mortality, disability, medical and productivity costs, resulting in the loss of about 16,782 quality-adjusted life years (Hoffmann et al., 2012; Foley et al., 2013; Mather et al., 2013).

*Salmonella* isolates can be differentiated into more than 2,600 serotypes based on their surface antigen profiles. The most common serotypes associated with human infections in the United States include Typhimurium, Enteritidis, Newport, Javiana, and Heidelberg (Foley and Lynne, 2008; Foley et al., 2013). These serotypes have shown the ability to infect multiple host species while other serotypes have a narrow host range; for example, *Salmonella* Typhi and *Salmonella* Paratyphi are human-associated serotypes that typically are not associated with foodborne transmission (Lynne et al., 2009; Foley et al., 2013). According to data from the United States Centers for Disease Control and Prevention (CDC) Food Net Program, greater than half of the human infections involving narrow host range serotypes caused invasive infection, rather than gastroenteritis, which leads to more severe disease outcomes (Vugia et al., 2004; Foley et al., 2013).

Over the past several decades, the incidence of multidrug-resistant (MDR) *Salmonella* infections has exhibited a steady rise in many regions including Europe and North America (Helms et al., 2005) and they have been associated with a number of outbreaks in the United States (Nair et al., 2018). Several studies have indicated that *S. enterica* resistance to antimicrobial drugs are correlated with an increased need for hospitalization, higher risk of invasive illness and deaths, as well as increased treatment costs (Helms et al., 2005; Mather et al., 2013). Based on epidemiological data, the CDC estimates the incidence of *Salmonella* infections per 100,000 population ranged from 11 to 15 during the years of 1996 to 2011 (Boore et al., 2015). There were 91,408 confirmed clinical cases of *Salmonella* foodborne illnesses in the European Union in 2014 (Pornsukarom et al., 2018).

## The Rise of AMR

**Figure 1:**
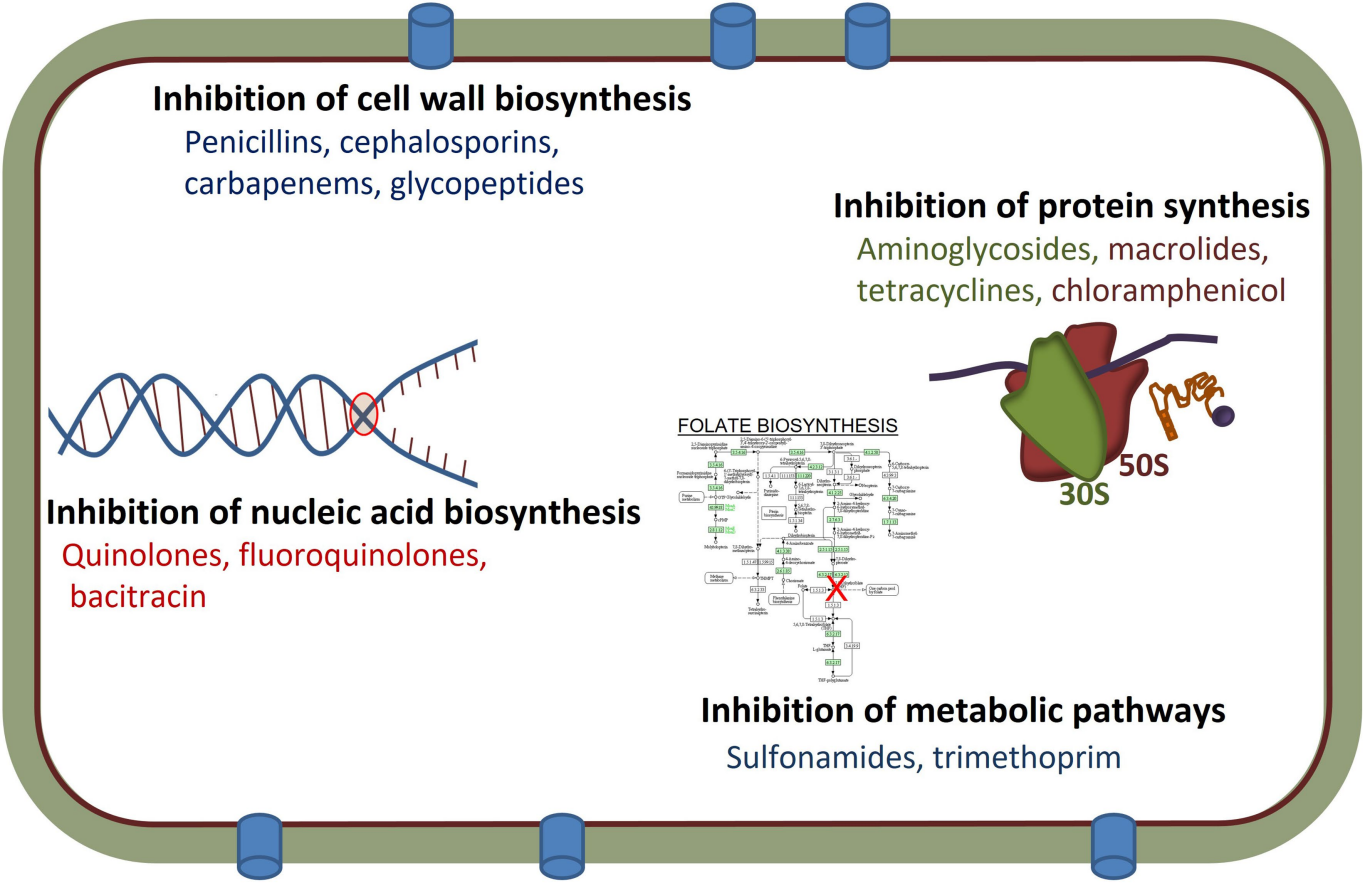
Mechanisms of actions and targets of different antimicrobial agents used in human and/or veterinary medicine.

Over the past several decades, antimicrobial agents have been a cornerstone of modern medicine and used extensively in animal production, veterinary medicine and industrial production (van Schaik, 2015). Antimicrobial therapies target specific aspects of bacterial physiology which selectively impacts the pathogens, while having less deleterious effects on the vertebrate host, but potentially can impact the commensal microbiome of the host (Jernberg et al., 2010). Antimicrobial agents are categorized based on multiple features involving their structure, ranges of target organisms (spectrum of activity), and mode of action. Most act by specifically binding to their targets, which plays a vital role in bacterial growth and survival, thereby preventing the physiological function of these targets and becoming lethal to the bacterial cells or inhibitory to cell growth (Lambert, 2005). The efficacy of an antimicrobial is related largely to its mechanisms of action, which include: (i) the inhibition of cell wall biosynthesis (penicillins and other β-lactams); (ii) protein synthesis inhibition by the targeting of the 16S rRNA (A-site) of the 30S ribosomal subunits (tetracyclines and aminoglycosides) or prevention of the 50S ribosomal subunit function (macrolides and chloramphenicol); (iii) inhibition of nucleic acid biosynthesis including inhibition of RNA transcription (rifampicin) or inhibition of DNA synthesis (quinolones and fluoroquinolones); (iv) inhibition of the metabolic pathways (including folic acid analogs such as sulfonamides and trimethoprim); and (v) by damaging the bacterial cell membrane structure (polymyxins; Bockstael and Van Aerschot, 2009; Sultan et al., 2018; Figure 1). As a result of their therapeutic use, antimicrobial agents have led to increased human life expectancy and decreased human morbidity and mortality (van Hoek et al., 2011).

However, due to overuse or misuse of the antimicrobials, antimicrobial resistance (AMR; a situation in which the drugs are unable to inhibit bacterial growth and lose the ability to be used for treating a bacterial infection) has emerged worldwide, and has become a major public health concern (Blair et al., 2015; Ventola, 2015; Zaman et al., 2017). In the United States, MDR bacteria infections have led to increased mortality and a strained health care system, with an estimated economic impact of over $20 billion associated with more than two-million infections and approximately 23,000 deaths each year (Blair et al., 2015).

**Figure 2:**
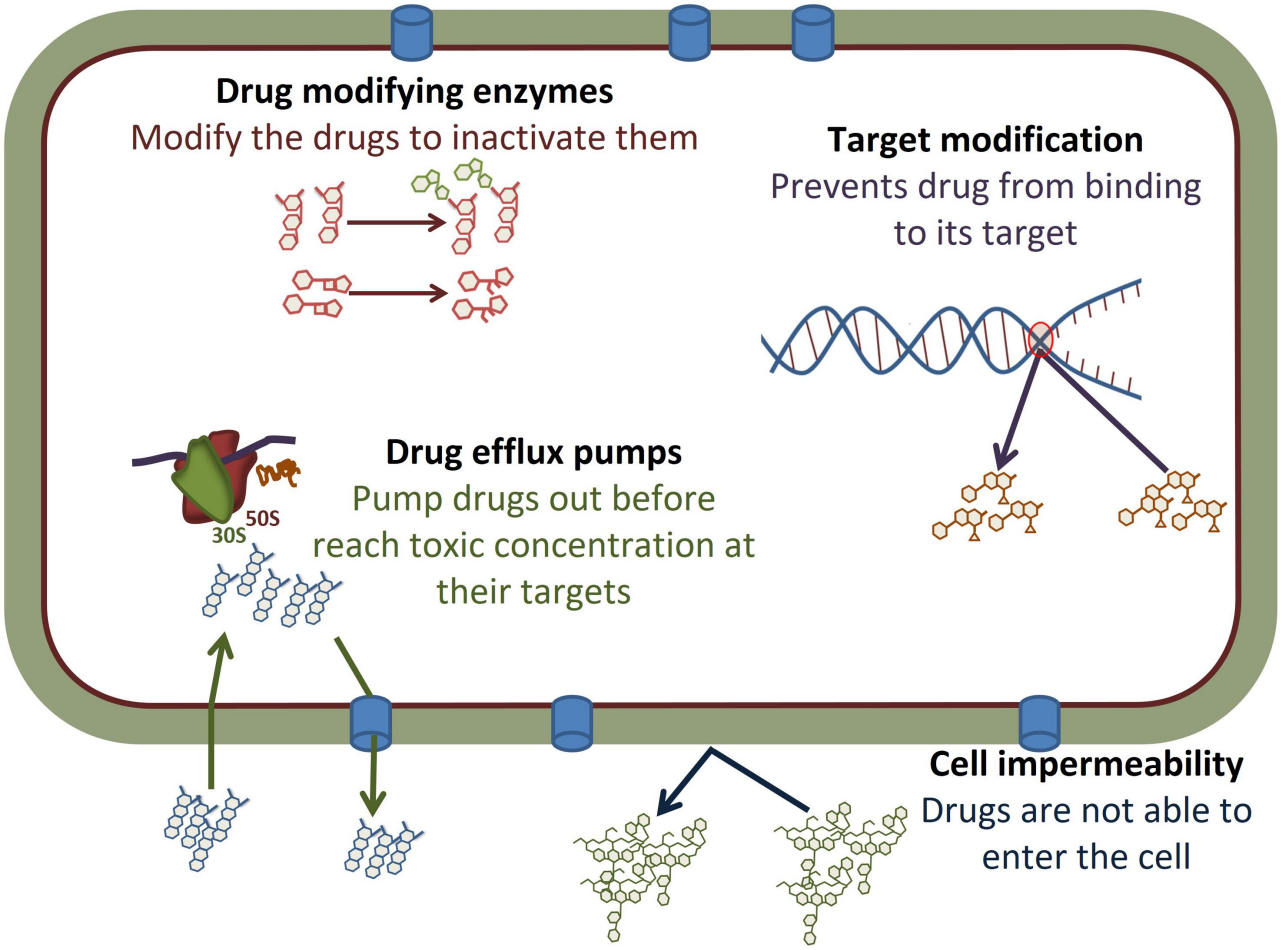
Antimicrobial resistance mechanisms observed in *Salmonella*. The different mechanisms are described in detail in the body of the manuscript. Briefly, drugs such as many aminoglycosides can be modified by the addition of functional groups, while others such as β-lactam antibiotics can be cleaved by enzymes, thus modifying their structures and inhibiting function. Target modification, such as can occur in metabolic pathways or in the nucleic acid replication machinery, can limit the ability of antimicrobials to bind to their targets to prevent efficacy. A number of other drugs are impacted by drug efflux pumps that prevent drugs such as tetracyclines and chloramphenicol from reaching their targets, such as the ribosomes, in a high enough concentration which limits their effectiveness. Other drugs, such as vancomycin, are too large to enter through the Gram-negative cell wall and *Salmonella* are intrinsically resistant due to inability to enter the cell.

The mechanisms of acquired resistance to antimicrobial agents are broadly classified into three categories: (i) use of energy-dependent efflux pumps to extrude the antimicrobials; (ii) production of hydrolytic or modifying enzymes to inactivate antimicrobials; and (iii) modifying antimicrobial targets (Blanco et al., 2016; Reygaert, 2018; Figure 2). In addition, many Gram-negative bacteria are naturally resistant to some larger antimicrobials, such as vancomycin, due to cell membrane characteristics that prevent entry into the cell and subsequent effectiveness.

Efflux pumps are bacterial transport proteins that are encoded by genes located on the bacterial chromosome and/or plasmids. The efflux pumps function primarily to extrude substrates (antimicrobials) from the cellular interior to the external environment, therefore imparting the efflux pump-expressing bacteria with an AMR phenotype. While some efflux pumps are expressed constitutively, others are induced under certain environmental stimuli or when a suitable substrate is present (Blanco et al., 2016). Many of these efflux pumps can transport a large variety of compounds (MDR efflux pumps), resulting in the acquisition of MDR by bacteria. In order to transport substrates against a concentration gradient, these efflux pumps are energy-dependent. Based on the type of the energy used, the efflux pumps are broadly classified into two categories, the primary efflux pumps which utilize energy from active hydrolysis of ATP and the secondary efflux pumps which derive energy from chemical gradients formed by either protons or ions such as sodium (Sharma et al., 2019).

In prokaryotes, five major families of efflux pumps have been described: (i) ATP binding cassette (ABC), which are primary active transporters; (ii) small MDR family; (iii) multidrug and toxin extrusion (MATE) family; (iv) major facilitator superfamily (MFS); and (v) resistance nodulation cell division (RND) family, which are all secondary active transporters (Sharma et al., 2019). Based on sequence similarity, the nine functional drug efflux pumps identified in *S. enterica* (AcrAB, AcrD, AcrEF, MdtABC, MdsAB, EmrAB, MdfA, MdtK, and MacAB) either belong to the MFS family (EmrAB and MdfA), the RND family (AcrAB, AcrD, AcrEF, MdtABC, and MdsAB), the MATE family (MdtK); or the ABC family (MacAB), respectively (Horiyama et al., 2010). Among these efflux pumps, the AcrAB-TolC efflux pump (a tripartite complex consisting of a periplasmic membrane fusion protein AcrA, a cytoplasmic membrane transporter protein AcrB, and an outer membrane channel TolC) is the most effective in causing MDR *Salmonella* and has been shown to directly contribute to resistance to fluoroquinolones, chloramphenicol, and tetracyclines in *Salmonella* (Piddock, 2006; Horiyama et al., 2010; Shen et al., 2017). The OqxAB efflux pump also mediates MDR in various bacteria, including *Salmonella* (Aljahdali et al., 2019). It belongs to the RND family and significantly contributes to reduced susceptibility to olaquindox, nalidixic acid, tigecycline, nitrofurantoin, chloramphenicol, and facilitates the development of high-level fluoroquinolone resistance (Li et al., 2013; Aljahdali et al., 2019). The *oqxAB* gene is located either on the chromosomal DNA of *Salmonella* (Wong and Chen, 2013) or on plasmids with other AMR genes (Li et al., 2013; Wong et al., 2016). The carriage of *oqxAB* on transferable plasmids would facilitate its transmission *via* horizontal gene transfer and the emergence of MDR strains (Wong et al., 2016; Aljahdali et al., 2019). Plasmids from the incompatibility group (Inc) HI2 have been shown to play a pivotal role in dissemination of *oqxAB* in *Salmonella* spp. (Wong et al., 2016).

The resistance to antimicrobials that target the ribosomal subunits interferes with the ability to bind to the ribosome. This binding interference is due to ribosomal mutation (aminoglycosides and oxazolidinones), ribosomal subunit methylation (aminoglycosides, macrolides, oxazolidinones, and streptogramins) most commonly involving *erm* genes, or ribosomal protection (tetracyclines; Reygaert, 2018). The tetracycline family of antimicrobials inhibits protein synthesis by preventing the attachment of aminoacyl-tRNA to the ribosomal acceptor (A) and is widely used because it offers a broad spectrum of activity against Gram-positive and Gram-negative bacteria. However, the increasing incidence of resistance to tetracyclines in *Salmonella* spp. of human and animal origins has been reported worldwide. In addition to efflux pump-mediated resistance mechanisms, resistance to tetracyclines can involve ribosomal protection proteins (RPPs), which are a group of cytoplasmic proteins that can bind to the ribosome, resulting in the release of tetracycline from the ribosome, enabling protein synthesis to proceed (Warburton et al., 2016). To date, 12 tetracycline resistance genes encoding RPPs have been reported, including *tet*M, O, Q, S, T, W, 32, 36, 44, *B*(P), *otr*(A), and *tet* (Warburton et al., 2016). Many of the RPP determinants are located on mobile genetic elements (MGEs) within *Salmonella*, including transposons or plasmids, which may have facilitated their spread throughout the eubacteria *via* lateral gene transfer events (Gargano et al., 2021). Another example of modification of antimicrobial targets is the wide dissemination of the plasmid-encoded chloramphenicol-florfenicol resistance (cfr) methyltransferase, which specifically methylates the adenine at position 2,503 in the 23S rRNA, thereby conferring resistance to a wide range of ribosome-targeted antimicrobials, including the phenicols, streptogramins, macrolides, and oxazolidinones (such as linezolid; Kaminska et al., 2010).

For antimicrobials that target nucleic acid synthesis (for example, fluoroquinolones), resistance is primarily associated with chromosomal mutations in the bacterial genes encoding targeted enzymes, DNA gyrase (such as *gyrA* in Gram-negative bacteria, including *Salmonella*) or topoisomerase IV (such as *grlA* in Gram-positive bacteria or *parC* in Gram-negative bacteria). Many of these mutations occur in the quinolone resistance determining region (QRDR) of the gyrase and topoisomerase genes and cause changes in the structure of the respective proteins that decreases or eliminates the ability of the antimicrobials to bind to the enzymes (Lee et al., 2021). The emergence of fluoroquinolone-resistant *Salmonella* has resulted in treatment failure and high mortality rates (Lee et al., 2021).

Antimicrobials such as the sulfonamides and trimethoprim act as competitive inhibitors of essential steps in the folate biosynthetic pathway in bacteria. These drugs are structural analogs of the natural substrates (sulfonamides for p-amino-benzoic acid and trimethoprim for dihydrofolate) and bind to their respective enzymes through competitive inhibition by binding to the active site of the enzymes dihydropteroate synthase and dihydrofolate reductase, respectively. Resistance to these antimicrobial that inhibit metabolic pathways can be due to mutations in enzymes, such as dihydropteroate synthase or dihydrofolate reductase, that prevent binding of the competitive inhibitor (Reygaert, 2018). Mutations in these enzymes at or near the active site result in structural changes in the enzyme that interfere with antimicrobial binding, while still allowing the natural substrate to bind (Reygaert, 2018). Additionally, overproduction of dihydrofolate reductase or dihydropteroate synthase can limit the competitive impact of sulfonamides and trimethoprim on the pathway function (Reygaert, 2018).

Antimicrobial inactivation by enzymes is another critical resistance mechanism. There are two main ways in which bacteria inactivate drugs, these include actual degradation of the drug or by the transfer of a chemical group (most commonly acetyl, phosphoryl, and adenyl groups) to the antimicrobials altering their function (Reygaert, 2018). The β-lactamases are a very large group of drug hydrolyzing enzymes that inactivate β-lactam drugs (including penicillin, ampicillin, cephalosporins, carbapenems, and ceftazidime) by hydrolyzing a specific site in the β-lactam ring structure, causing the ring to open, resulting in the inability of the antimicrobials to bind to their target penicillin-binding protein (PBP). To date, more than 1,300 distinct β-lactamases have been identified in clinical isolates, of which the most deleterious are the extended-spectrum β-lactamases (ESBLs) that hydrolyze most penicillins, cephalosporins, and the carbapenemases that can inactivate all β-lactam classes of drugs (Bush, 2013). The production of β-lactamases, which can be encoded by genes located on the chromosome (such as *bla*_SHV-12_, *bla*_CTX-M-9a_, and *bla*_MIR_) or on plasmids (for example, *bla*_TEM_ or *bla*_CTX-M-15_), is the most common resistance mechanism used by Gram-negative bacteria against β-lactam drugs (Bush and Bradford, 2016; Reygaert, 2018). Combined with decreased uptake or increased efflux of the drugs, resistance to the β-lactams in Gram-negative bacteria continues to be on the rise with the high-level resistance being a major clinical problem (Bush and Bradford, 2016).

Inactivation by enzymatic modification is also the most prevalent mechanism of resistance to aminoglycoside antimicrobials, which is currently considered to be one of the more formidable broad-spectrum antimicrobials used in the treatment of life-threatening infections in the clinical setting (Ramirez and Tolmasky, 2010; Aishwarya et al., 2020). Aminoglycoside-modifying enzymes (AMEs) are the most common cause of resistance to aminoglycosides (Ramirez and Tolmasky, 2010). AMEs catalyze the covalent modification of aminoglycosides as they transport across the cytoplasmic membrane by modifying the amino or hydroxyl groups. There are three kinds of AMEs: (1) N-acetyltransferases (AAC), which acetylate the –NH2 (amino) group by N-acetylation; (2) O-nucleotidyltranferases (ANT), which adenylate the hydroxyl groups by O-nucleotidylation; and (3) O-phosphotranferases (APH), which phosphorylate the hydroxyl groups by O-phosphorylation (Ramirez and Tolmasky, 2010). The modifications of the aminoglycoside reduces drug binding to the ribosome, which results in high levels of resistance. The genes coding for AMEs are highly mobile with the ability to transfer as part of integrons, gene cassettes, transposons, integrative conjugative elements, or through conjugation as part of mobilizable or conjugative plasmids (Ramirez and Tolmasky, 2010). To date, more than 85 AMEs have been reported in both Gram-positive and Gram-negative bacteria (Aishwarya et al., 2020). The combination of large numbers, the ability of the genes coding for these enzymes to evolve, as well as the numerous mobile elements where they are located, result in a high adaptability AMEs to efficiently disseminate among bacteria, which has greatly reduced the efficacy of several aminoglycosides (Ramirez and Tolmasky, 2010). Tetracyclines can also be inactivated by hydrolyzation *via* the TetX enzyme, whose gene is located on plasmid that catalyzes tetracycline degradation (Moore et al., 2005).

Antimicrobial inactivation by the transfer of a chemical group is another key resistance challenge, as a large number of transferases that have been identified contribute to resistance. Among them, acetylation is the most diversely used mechanism and has led to resistance against aminoglycosides, chloramphenicol, streptogramins, and fluoroquinolones (Reygaert, 2018). The primary resistance mechanism for chloramphenicol is the enzymatic inactivation by acetylation of the antimicrobial drug molecule *via* different types of chloramphenicol acetyltransferases (CATs; Schwarz et al., 2004). There are two different types of CAT enzymes which are genetically unrelated and encoded by *catA* and *catB* groups, respectively (Roberts and Schwarz, 2017). Both *catA* and *catB* genes are often associated with mobile elements such as plasmids, transposons, or gene cassettes and are able to be transferred between bacteria of different species and genera (Roberts and Schwarz, 2017). In *Salmonella*, besides mutations in the QRDR and plasmid-mediated quinolone resistance (PMQR) determinants *qnr* genes; *aac(6′)-Ib-cr* encodes acetyltransferase which can acetylate the quinolone antimicrobials that reduces their activities and contribute to quinolone resistance (Robicsek et al., 2006).

The detection of AMR genes in bacteria has mainly relied on molecular methods. Due to their rapidity and sensitivity, nucleic acid-based detection methods have played an important role in the elucidation of resistance mechanisms (Fluit et al., 2001). Based on the amplification of target genes, traditional PCR, multiplex PCR, qPCR (real-time PCR), Reverse Transcriptase PCR (RT-PCR), approaches have been developed and widely used in the laboratory to rapidly identify AMR genes (Galhano et al., 2021). DNA microarray and hybridization approaches have also been used to simultaneously detect a large number different AMR genes in a short time. Microarrays for resistance detection in both gram-negative and gram-positive bacteria have been developed (Perreten et al., 2005; Card et al., 2013; Galhano et al., 2021). Microarray detection methods have been largely replaced by whole-genome sequencing (WGS) approaches that have become widely used and effective tools in detection of AMR genes (Galhano et al., 2021). To detect AMR genes in WGS data, several AMR gene databases, such as ARG-ANNOT (Antibiotic Resistance Gene-ANNOTation), RGI/CARD (Comprehensive Antibiotic Resistance Database), NCBI-AMRFinder, and PointFinder have been developed to identify AMR genes in the WGS contigs (Gupta et al., 2014; Zankari et al., 2017; Alcock et al., 2020; Feldgarden et al., 2021). WGS has been proven to be an effective technique to detect AMR genes within bacteria such as *Salmonella* (Galhano et al., 2021).

### Mobilome Among *Salmonella enterica* Isolates

The term “mobilome” refers to all MGEs and genes including plasmids, integrons, transposons, insertion sequences (ISs), integrative and conjugative elements (ICEs), and resistance islands and/or genomic islands (GIs) in bacteria which can translocate within and transfer between genomes (Frost et al., 2005; Cabezón et al., 2017). MGEs play a key role in both the development and spread of AMR genes among the bacterial populations as well as in the evolution of bacterial genomes which rapidly respond to a selective pressure. These responses include alteration of antimicrobial exposure in different genera, species, and strains, including those of *Salmonella* isolates (Davies and Davies, 2010).

## The Mobility of the AMR Genes Through MGEs

### Plasmids

Understanding the transfer dynamics of AMR plasmids requires detailed monitoring of different pathogenic bacteria in both clinical and non-clinical environments. Plasmid-mediated transfer plays a critical role in the spread of AMR in *S. enterica* and related species (Han et al., 2012; San Millan et al., 2015; San Millan, 2018). Several studies and review articles have described the importance of plasmid dynamics in Gram-negative bacteria, in particular *S. enterica*, and many have utilized DNA sequencing to help examine resistance transfer (Zhao et al., 2003; Han et al., 2012; Lerminiaux and Cameron, 2019; McMillan et al., 2019). Baker et al. (2018) highlights the importance of WGS as a key technology for understanding the spatial dynamics of AMR evolution and for genomic epidemiology (Baker et al., 2018). Tables 1, 2 present multiple sequencing platforms and computational-based approaches that can be used for the detection of plasmid-based AMR genes.

**Table 1:** Approaches to detect plasmid-based antimicrobial resistance (AMR) genes.

Approach	Sample types	Advantage	Limitation	References
Illumina-based WGS	Food/clinical samples	Fast, applicable for studying the genetic diversity evolutionary history of plasmid type and epidemiological tracking.	Challenge for plasmid detection in short read length.	Almeida et al., 2018
Oxford Nanopore Technology (ONT)	Food/clinical samples	Rapid tool Low instrument Cost and small footprint	Incomplete fragmentation of plasmid. High error rate. Performance often needs to be optimized with MiSeq reads.	Lemon et al., 2017
Pacific bioscience single-molecule real-time sequencing (SMRT)	Food/clinical samples	Generate long-read sequencing data that can lead to closed plasmid sequences.	The reads length of data relies on the individual polymerase.	Chen et al., 2019

**Table 2:** *In silico* analysis of resistance plasmid gene content and epidemiology.

Web server	Advantages	Links	References
PLSDB	Used for short sequences to identify the specific AB genes in the plasmids.	https://ccb-microbe.cs.uni-saarland.de/plsdb/	Galata et al., 2019
ATLAS	Detect the plasmids carrying the antimicrobials resistance genes, and plasmid network.	http://www.patlas.site	Jesus et al., 2019
PlasmidSeeker	Able to identify the novel plasmids and detect plasmids from bacterial WGS data without read assembly.	https://github.com/bioinfo-ut/PlasmidSeeker	Roosaare et al., 2018
FeatureExtract	Able to identify the antimicrobial resistance genes and conjugal transfer system (IV).	https://services.healthtech.dtu.dk/service.php?FeatureExtract-1.2	Wernersson, 2005

In studies of MDR plasmid transfer in different *Salmonella* strains, isolates have been sequenced and diverse AMR genes associated with chloramphenicol, tetracyclines, ampicillin, streptomycin, kanamycin, and β-lactam antimicrobials have been identified (Han et al., 2012). When the rate of horizontal transfer outweighs the costs they impose on their bacterial hosts, plasmids are favored in the new hosts (Svara and Rankin, 2011). Stalder et al. (2017) reported that common mechanisms of plasmid stabilization in bacterial pathogens could influence plasmid-host coevolutionary dynamics (Stalder et al., 2017). Various concentrations of antimicrobials are encountered by bacteria in different environments and may affect the dynamics of the microbial population. For example, plasmid transfer between bacteria may lead to acquired resistance to different antimicrobials through the transfer of AMR determinants in response to selective pressure (Martinez, 2008). Svara and Rankin (2011) designed a model to examine the effect of antimicrobial treatment, specifically the dosage of antimicrobials and the interval between treatments, on the evolution of plasmid-borne resistance (Svara and Rankin, 2011). Their results showed that different treatment regimens (different interval between antimicrobial treatments and the dosage of antimicrobials) can select for either plasmid-carried or chromosome-carried resistance. In the absence of competing non-resistant plasmids, high transmission favors plasmid-borne resistance. While in the presence of other plasmids, plasmid-borne resistance was favored over chromosomal resistance in the low frequency antimicrobial treatments, but a high intensity of antimicrobial treatments is required for resistance plasmids to outcompete non-resistance plasmids (Svara and Rankin, 2011). Therefore, changes in natural ecosystems, including the release of large amounts of antimicrobials, might alter the population dynamics of antimicrobial resistant microorganisms, which will affect the evolution and dissemination of AMR in nature (Martinez, 2008). Taken together, these findings emphasize the necessity of new therapeutics to reduce the spread of AMR.

According to their transmissibility by conjugation, there are two classes of transmissible plasmids: (1) conjugative plasmids, which contain a full set of conjugation genes; and (2) mobilizable plasmids, which contain only a minimal set of genes that allow them to be mobilized by conjugation when they coexist in the same donor cell with a conjugative plasmid (Garcillan-Barcia et al., 2011). Conjugative plasmids possess a generally low copy number, while mobilizable plasmids tend to be a high copy number (Watve et al., 2010; van Hoek et al., 2011). Both conjugative and mobilizable plasmids can encode AMR genes and transfer them to a new host (van Hoek et al., 2011). Over the last decade, more than 1,000 unique plasmids have been identified in the family of *Enterobacteriaceae*, some of the most common ones include IncA/C, HI2, HI1, I1-γ, X, L/M, N, FIA, FIB, FIC, W, Y, P, T, K, B/O, FI1, U, R, ColE, and Q (Garcia-Fernandez and Carattoli, 2010). Among these, some Inc. types can be clustered into groups based on the genetic and pilus structure: (1) IncF group (consisting of IncF, D, J, and S); (2) IncI group (includes IncB, I, and K); and (3) IncP group (containing IncM, P, U, and W); and Ti (tumor inducing) that may have implications on plasmid transfer dynamics (van Hoek et al., 2011). Specifically, many of the plasmids that have been identified in *Salmonella* strains include representatives of IncA/C, F, H, I, L/M, N, R, and X groups (Fricke et al., 2009; Foley et al., 2013).

In the 1970s, IncA/C plasmids were initially described as large-molecular-weight (140–200 kb) and low copy number plasmids present in fish pathogens such as *Aeromonas hydrophila* and *Vibrio* spp. (Carraro et al., 2015; Ruggiero et al., 2018). Subsequently, IncA/C plasmids were found to encode resistance genes to antimicrobial agents in food animals and agricultural settings (Johnson and Lang, 2012). To date, research on conjugative IncA/C plasmids has determined that these plasmids play an essential role in the spread of MDR among the species of *Enterobacteriaceae*, including *S. enterica* and *Vibrio cholerae*, which is an important concern in the public health community (Carraro et al., 2015; Han et al., 2018). The core genetic backbones of IncA/C plasmids are generally highly conserved, with greater than 98% nucleotide identity across the plasmid backbone. Most examples of IncA/C plasmids among non-typhoidal *S. enterica* isolates in the United States carry transfer-associated genes which accelerate conjugal transfer (Welch et al., 2007; Fricke et al., 2009; Wiesner et al., 2011; Han et al., 2018; Ruggiero et al., 2018). Wiesner et al. (2011) described a MDR *Salmonella* Typhimurium ST213 strain in Mexico, which was associated with the carriage of IncA/C plasmids that carried a plasmid-borne *bla*_CMY-2_ gene encoding resistance to extended-spectrum cephalosporins (Wiesner et al., 2011). As predicted by Han et al. (2018), many IncA/C plasmids encode their own conjugal machinery and are able to be transferred within bacterial communities. However, others without the full cohort of transfer genes required other conjugative plasmids in the bacterial cell for transfer of the plasmids (Han et al., 2018). Rankin et al. (2002) indicated that IncA/C plasmids were responsible for MDR phenotypes in *Salmonella* Newport isolates and their resistance genes could be transferred both to other *Salmonella* as well as other susceptible organisms under antimicrobial selection pressure in the same environments (Rankin et al., 2002). In *S. enterica* serovar Newport isolates, IncA/C plasmids emerged as the source of MDR phenotypes after 1980 (Johnson and Lang, 2012). Moreover, these plasmids are considered as broad host range plasmids due to their ability to spread by conjugative transfer among the bacterial communities, meanwhile under *in vitro* conditions some IncA/C plasmids exhibit variation in their ability to transfer between *Escherichia coli* and *S. enterica* (Johnson and Lang, 2012). In addition to their presence in food animal environments, cases have also been documented of plasmids spreading in hospital environments (Johnson and Lang, 2012). In some of these studies it was demonstrated that IncA/C plasmids have mobilizable genes conferring resistance to a number of different antimicrobial agents including β-lactams, aminoglycosides, chloramphenicol, sulfonamide, trimethoprim, quinolones, and tetracyclines (Carraro et al., 2015). These studies have been somewhat hampered by the presence of a large number of AMR elements carried on the plasmids. These AMR elements have been a challenge for studying their basic molecular biology and regulatory mechanisms because most of the molecular biology tools that are available rely on the antimicrobial selection for mutational studies (Johnson and Lang, 2012; Carraro et al., 2015).

#### Conjugative Plasmids

One of the main mechanisms of the horizontal gene transfer is bacterial conjugation (Frost et al., 2005; Cabezón et al., 2017). Bacterial conjugation is a highly specific process that occurs by transferring plasmid DNA from one bacterial cell (donor) to another bacterium (recipient) through a direct cell-to-cell contact (Willetts and Wilkins, 1984; Partridge et al., 2018). Conjugative plasmids are considered to be self-transmissible and carry the genes, known as mobilization (MOB) or DNA-transfer replication (Dtr) genes, that are required for DNA transfer and mating-pair apparatus formation to initiate the transfer process at OriT (Jain and Srivastava, 2013). The MOB functionality has also been used for the identification/classification/typing of plasmids and epidemiological tracking based on the conjugative and mobilization relaxase genes (Partridge et al., 2018). The fundamental principle of the bacterial conjugation system involves the merging of the two archaic bacterial systems: rolling-circle replication (RCR) and the type IV secretion system (T4SS; Llosa et al., 2002). The T4SS forms a complex protein encoded by numerous genes called mating pore formation genes (MPF; Cabezón et al., 2017). In the conjugation machinery, RCR has a similar function to a relaxosome, which initiates transfer through the T4SS (Llosa et al., 2002). Howard et al. (1995) demonstrated that the two proteins (TraYp and TraLp) assemble the relaxosome site and bind at the origin of the transfer which causes a strand-specific nick found within conjugation machinery in the *E. coli* F plasmids (Howard et al., 1995). There are two basic regions that contribute to conjugation machinery: Tral, also known as TrhX, which carries OriT, relaxosome genes and some MPF components; and Tra2, or TrhZ, which consists of genes encoding MPF proteins (Partridge et al., 2018). It has been reported that the evolution of conjugation mobility systems, with their relaxases and type IV coupling protein (T4CPs) diverged from the prototypical T4SS (Smillie et al., 2010). Study in our lab demonstrated that some *Salmonella* strains with plasmids carrying a VirB/D4 T4SS survived better in epithelial cells and macrophages than those without the plasmid (Gokulan et al., 2013).

#### Mobilizable Plasmids

Unlike conjugative plasmids which are self-transmissible, mobilizable plasmids lack the necessary genes for complete conjugation and are therefore not self-transmissible (Francia et al., 2004). Mobilizable plasmids have an origin of transfer site (*oriT*), a region essential for replication, and a mobilization gene (*mob*). They have an ability to exploit conjugative plasmids for horizontal dissemination, but are non-mobile in cells that lack mobile elements carrying compatible mating-pore genes (Ramsay et al., 2016). Like conjugative plasmids, mobilizable plasmids in *Salmonella* also can carry AMR genes.

The IncQ1 plasmids are examples of mobilizable plasmids. They are small in size (10–12 kb) and have broad host range (McMillan et al., 2020). Generally, they are associated with AMR genes including *strAB*, *tetAR*, and *sul2*, and can be mobilized by large plasmids including the IncF, I1, N, P, W, or X plasmids (Francia et al., 2004; McMillan et al., 2020). Baker et al. (2008) demonstrated that a small region in *Salmonella* pathogenicity island 7 (SPI-7) in *Salmonella* Typhi was able to mobilize IncQ plasmid R300B (Baker et al., 2008). Several examples of the the IncQ plasmid (MoB_Q_) in different *Salmonella* strains are presented in Table 3. Other examples of mobilizable plasmids are the IncR plasmids (40–160 kb), which were first identified in 2009 from *Klebsiella* and *Salmonella* Montevideo (Garcia-Fernandez et al., 2009). IncR plasmids have been reported to carry genes conferring resistance to antimicrobials belonging to numerous classes including: β-lactams, sulphonamides, quinolones, aminoglycosides, tetracyclines, chloramphenicol, and trimethoprim (Rozwandowicz et al., 2018; Plumb et al., 2019).

**Table 3:** Examples of mobilizable IncQ plasmids identified in *Salmonella enterica* strains.

Plasmid names	Antimicrobial resistance genes	Size (bp)	*Salmonella enterica* serotype	References
IncQ1	*sul2*, *strAB*, and *tetAR*	10,867 bp	Reading	Miller et al., 2020
C0144011- and C01RNA1-like	*bla_TEM-1C_*	10,384 bp	Reading	Miller et al., 2020
P28321a-1	*aph(3′)-Ia*, *aph(6)-Id*, *bla_TEM-1_*, *aph(3″)-Ib*, *sul2*, *tetB*, and *aac(3)-IId*	219,745 bp	Typhimurium	Zhao et al., 2020
PSTU288-2	*sulII*, *strA*, *strB*, *tetA*, and *cat*	11,067 bp	Typhimurium	Hooton et al., 2014

### Integrons

Integrons are mobile genetic elements containing a site-recombination system that is able to integrate, express, and exchange specific DNA known as gene cassettes (Silva et al., 2012; Ravi et al., 2014). Gene cassettes are defined as the smallest mobile elements with an AMR gene; they are located between two recombination sites (*attI* and *attC* 59-base elements; Rodriguez et al., 2006; Gillings, 2014).

Generally, integrons contain three important major elements: (1) the integrase, which is a gene encoding integration into the host genetic backbone, (2) the recombination site, which serves as the primary site to capture the resistance gene cassettes, and (3) the promoter. Each of these elements is required for transcription and drives expression of gene cassettes (Rodriguez et al., 2006; Silva et al., 2012; Gillings, 2014). Integrons are divided into two different types: (1) mobile integrons (MIS), which carry a limited number of AMR gene cassettes (these include the class I integron) and are involved in the dissemination of AMR; and (2) chromosomal integrons (CIS), which have a different number of gene cassettes and are not involved in the dissemination of AMR (Domingues et al., 2012). Moreover, integrons are found in a wide diversity of clinical bacterial strains and environmental isolates in both chromosomes and within MGE (Gillings, 2013). The first discovered chromosomally-located superintegrons were identified in *V. cholerae* isolates (Ravi et al., 2014). These superintegrons are located on the chromosome and typically carry more than 20 gene cassettes with unknown function (Ravi et al., 2014). Integrons are immobile on their own and yet have been observed to transfer across numerous bacterial genomes (Gillings, 2014). Integrons are divided into five classes based on the type of gene capture mechanism, gene cassettes, and the similarity of sequence which encodes integrases to facilitate insertion of the target DNA (Gillings, 2013, 2014; Ravi et al., 2014). Each of the five different classes of integrons (class I–V) encodes a distinct integrase gene. The class I integrons play an important role in the spread of AMR genes and are the most-studied types from clinical samples (Gillings, 2013). They are referred to as broad host range as they have been found both in commensal and pathogenic bacteria (Ravi et al., 2014) and are widely found across different *S. enterica* isolates (Rodriguez et al., 2006).

Recent comparative studies described how integrons contribute to MDR phenotypes (Gillings, 2014; Ravi et al., 2014; Deng et al., 2015). The integron location within MGEs and the chromosome has a unique advantage for generating genomic and phenotypic diversity (Gillings, 2014). Three different classes of mobile integrons (class I, II, and III) can contribute to the MDR phenotypes (Mazel, 2006). For example, class I and class II integrons are associated with the bacteria from animals and have an impact on the human gastrointestinal microbiota by transmission of AMR through the food supply. The horizontal transfer of class I integrons to commensal organisms *via* conjugation facilitates the development and spread of resistant bacteria (de Paula et al., 2018; Miller and Harbottle, 2018). Class I integrons can also play a key role in the transmission of AMR in the aquatic environment and in fish pathogens, as well as in terrestrial pathogens such as *S. enterica*, *E. coli*, and other bacteria species (Miller and Harbottle, 2018). Evershed et al. (2009) discovered IncA/C plasmids, which play an important role in the dissemination of AMR genes, have an unusual cassette in their class I integrons, with most carrying the same set of resistance genes including: *aacU*, *aphA*, *hph*, *sul2*, and *tetA*(D) (Evershed et al., 2009). Similar integrons have also been identified in *S. enterica* serovars Senftenberg and Ohio. IncA/C plasmids have a broad host range, having been recovered from numerous species, their conjugal transfer capacities are variable (Evershed et al., 2009; Wiesner et al., 2013).

Moreover, Villa and Carattoli (2005) reported that some plasmids in MDR *S.* Typhimurium strains carry *sprC*, *rck*, and *pefA* virulence genes, in addition to two class I integrons carrying AMR genes within the Tn22 and Tn1696 transposons (Villa and Carattoli, 2005). Thus, it was shown that the association between MDR and virulence determinants may contribute to virulence plasmid evolution (Villa and Carattoli, 2005). Likewise, Rodriguez et al. (2006) reported that the class II integrons are present on self-transferable plasmids of *Salmonella* (Rodriguez et al., 2006). It is well-known, that the diversity of integrons plays a critical role in the spread of AMR genes and can drive evolution in *S. enterica*. Several integrons (both class I and class II) have been detected in clinical or environment isolates of *Salmonella* (Yeh et al., 2018) and other *Enterobacteriaceae* from different geographical locations (Adelowo et al., 2018; Gomi et al., 2018; Leungtongkam et al., 2018; Phoon et al., 2018).

### Transposons

Transposons, commonly referred to as “jumping gene systems,” provide functionality for the transfer of a segment of bacterial DNA to and from chromosomal or plasmid DNA and often carry AMR genes (Bennett, 2008; Giedraitiene et al., 2011; Sultan et al., 2018). Transposons are made up of a transposase and inverted repeat sequences on their ends. The inverted repeat sequences can accelerate target recognition and recombination; while the transposase catalyzes the movement of DNA segments to another part of the genome by a cut-and-paste mechanism, or by a replicative transposition mechanism, and functional genes (such as AMR genes; Giedraitiene et al., 2011). Furthermore, transposable elements (TEs) consist of a set of mobile elements that include small cryptic elements, IS elements, transposons, and transposing bacteriophages. The bacteriophage is a bacterial virus, which is also known as a transposing phage, that infects and replicates its own sequences within bacteria through either a lysogenic or lytic cycle (Balcazar, 2014). In the lysogenic cycle, the phage is referred to as temperate or non-virulent, and occurs following the injection of bacteriophage DNA into the bacteria cell. During this phase, the bacteriophage integrates its genome into the host genome *via* the phage-encoded integrases to form prophage, where it replicates passively as part of the host genome. As the phage excises from the host chromosome, it may transport bacterial genes from one bacteria strain to another *via* a process called transduction (Bennett, 2008; Giedraitiene et al., 2011). In the lytic cycle, also referred to as virulent infection, immediately after injecting into the host cell, the bacteriophage genome synthesizes what are termed early proteins instead of integrating its DNA into the host genome. These early proteins function to break down the host DNA allowing the bacteriophage to control the host’s cellular machinery to synthesize the proteins required to build new phage particles. Eventually, the destruction of the infected bacteria cell results in the release of the new phage progeny. Transduction mediated by temperate bacteriophages can serve as a horizontal gene transfer mechanisms and has played an important role in the dissemination of AMR genes among bacterial populations (Bennett, 2008; Giedraitiene et al., 2011; Colavecchio et al., 2017). Multiple bacteriophage and prophages have been reported in *Salmonella* strains, such as Fels-1, Gifsy-2, P22, and FelixO1 (Switt et al., 2013; Colavecchio et al., 2017). Among them, *Salmonella* phage P22 has been widely used in molecular biology for its ability to introduce foreign genes, including AMR genes into recipient cells by transduction (Switt et al., 2013). Since its emergence in the 1990s, the MDR ACSSuT-type (resistance to ampicillin, chloramphenicol, streptomycin, sulfonamide, and tetracycline) *S.* Typhimurium phage type DT104, has become widely distributed worldwide. Studies showed that the ACSSuT-type region of DT104 was derived from two separate evolutionary events. One was the integration of a 43-kb *Salmonella* genomic island (SGI) 1 that carries multiple AMR genes and the other is the integration of a P22-like phage into the chromosome to form prophage PDT17 or ST104 (Chiu et al., 2006). More information about the contribution of bacteriophages to the spread of AMR genes among foodborne pathogens can be found in a review article wrote by Colavecchio et al. (2017).

**Figure 3:**
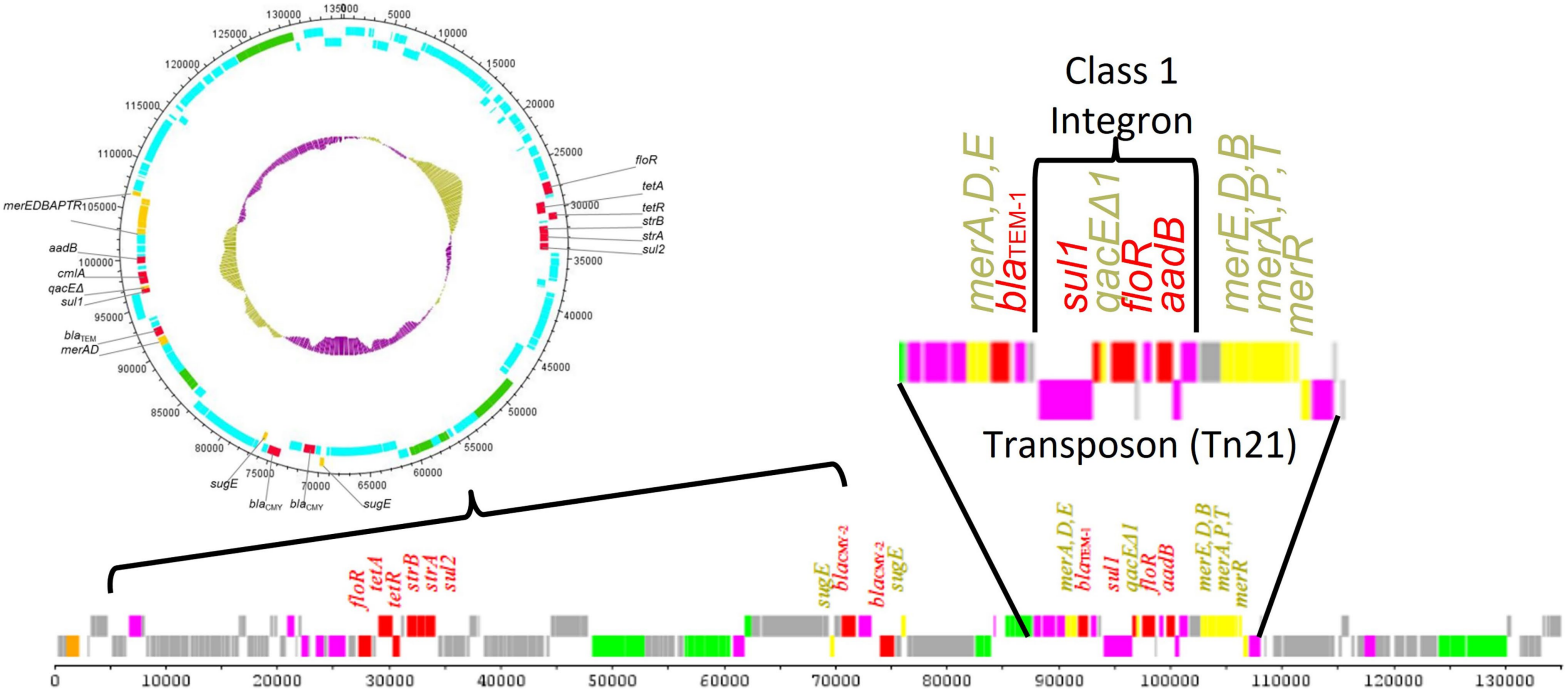
Results of the sequencing of a transmissible antimicrobial resistance plasmid from a *Salmonella enterica* strain (Han et al., 2012). The plasmid contains multiple antimicrobial resistance (denoted in red) and heavy metal/disinfectant (denoted in yellow) resistance genes, including those carried in a Tn21 transposon. Within the Tn21 transposon, there was a class 1 integron carrying multiple antimicrobial resistance genes. This nesting of MGEs within other MGEs provides significant opportunities for the generation and dissemination of diverse antimicrobial resistance elements. AMR regions and mobile genetic elements also often have a higher G + C content compared to the plasmid backbone as is highlighted in the inner ring of the circular plasmid diagram (gold vs. purple bars).

The sizes of transposon sequences range from 3 to 40 kb depending on the variability of the genes present in the transposons (Pagano et al., 2016). Figure 3 shows a representative transposon (Tn21) that is located within an IncA/C plasmid and carries a class 1 integron as well as multiple antimicrobial and disinfectant resistance genes (Han et al., 2012). There are two major types of TEs: (1) composite transposons (class I) and (2) complex transposons (class II). Class I transposons contain a variety of resistance genes and have little DNA homology in their central regions, which are flanked by IS elements (Pagano et al., 2016; Sultan et al., 2018). In *S. enterica* serovars, such as Typhimurium and Choleraesuis, class I transposons often reside in conjugative plasmids, such as IncHI2, and are responsible for AMR gene dissemination (Sultan et al., 2018). In contrast, class II transposons, constituting of three dissimilar, but interrelated families (Tn3, Tn21, and Tn2501), have more diverse genetic structures than class I transposons (Pagano et al., 2016; Sultan et al., 2018). A wide diversity of class I transposons (for example, Tn1696, Tn10, Tn6088, Tn3, Tn9, and Tn 1721-like transposon) carrying a variety of different AMR genes have been identified in *Salmonella* serovar (Frech and Schwarz, 1999; Orman et al., 2002; Cain et al., 2010; Cain and Hall, 2012; Chen et al., 2020). The TEs that are transferred include: (i) the integrative and conjugative elements (ICEs), (ii) the integrative and mobilizable elements (IMEs), and (iii) decayed elements derived from ICEs or IMEs, such as cis-mobilizable elements (CIMEs; Lao et al., 2020). CIMEs are decayed elements originating from ICEs or IMEs that have lost their integration and transfer genes, but retained *attL* and *attR* sites (Guedon et al., 2017). The CIMEs are known to be major vehicles for acquisition of a broad spectrum of AMR genes among bacteria and many other genes that can be advantageous for their hosts (Guedon et al., 2017). The recombination between related or unrelated ICEs, IMEs, and CIMEs, likely integrated in tandem, plays a major role in the evolution and plasticity of ICEs and IMEs (Bellanger et al., 2014; Lao et al., 2020).

#### Integrative and Conjugative Elements

Integrative and conjugative elements, also known as conjugative transposons, are genetic elements that are self-transmissible between chromosomes of different cells and are widely distributed among both Gram-negative and Gram-positive bacteria (van Hoek et al., 2011; Seth-Smith et al., 2012; Johnson and Grossman, 2015). Contrary to conjugative plasmids which are extrachromosomal DNA elements within a cell that are physically separated from the host chromosome and replicate independently, ICEs are typically found integrated in the host bacterial chromosome (Johnson and Grossman, 2015). They contain genes required for their integration and excision, through which they can excise from the host chromosome to form a circular product prior to conjugal transfer (Johnson and Grossman, 2015). Normally, the repression of transcription prevents the expression of the excision and conjugation genes and the ICEs are maintained as inert elements in the host chromosome (Johnson and Grossman, 2015). The de-repression/activation of expression of ICE genes under certain conditions lead to the induction of ICE genes, which in turn leads to their excision from the donor chromosome, formation of a circular intermediate, transfer by conjugation, and reintegrated into the recipient chromosome (Johnson and Grossman, 2015). In addition to an integrase and excisionase genes, ICEs also encode a functional type IV secretion system, which facilitates the conjugative transfer (Johnson and Grossman, 2015). While integrated in the chromosome, ICEs are passively propagated during chromosomal replication, segregation, and cell division (Johnson and Grossman, 2015). Each ICE also contains a variety of cargo genes that confer different phenotypes to host cells, for example, virulence determinants, antimicrobial-resistant factors and/or genes coding for other beneficial traits, indicating they play important roles for bacterial evolution (Arai et al., 2019). A well-known example is the *Salmonella* pathogenicity island (SPI)-7, which possesses features indicative of an ICE and carries genes implicated in virulence and has been found within some pathogenic strains of *S. enterica* (Seth-Smith et al., 2012). Seth-Smith et al. (2012) identified and analyzed ICEs related to SPI-7 within the genus *Salmonella* and other *Enterobacteriaceae* and discovered two new ICEs with high levels of identity to SPI-7, which indicates that these elements may be more common than previously thought in the *Salmonella* (Seth-Smith et al., 2012). They also identified more distantly related ICEs, with distinct cargo regions in other strains of *Salmonella* and members of the *Enterobacteriaceae* (Seth-Smith et al., 2012).

*Salmonella* genomic island 3 has been demonstrated to be an ICE and increases copper and arsenic tolerance in *Salmonella* strains (Arai et al., 2019). ICEkp, an ICE in *Klebsiella pneumoniae*, mobilizes a *ybt* locus which encodes biosynthesis of the siderophore yersiniabactin and its receptor. Yersiniabactin and other siderophore systems are key bacterial virulence factors as they provide mechanisms for scavenging iron (an essential nutrient) from host transport proteins, thereby enhancing the ability of bacteria to survive and replicate within the host (Lam et al., 2018). SXT/R391 family of ICEs, that is mainly associated with *Vibrio* spp., is one of the largest of the ICE families with more than 100 elements (Marrero and Waldor, 2007; Ryan et al., 2016). These elements integrate into an *attB* site in the 5′ end of the chromosomal *prfC* gene by site-specific recombination with their *attP* site, catalyzed by the IntSXT tyrosine recombinase (Partridge et al., 2018). ICEs of SXT/R391 family are able to mobilize adjacent sequences and have been recognized as major drivers of the dissemination of AMR genes among several species of *Enterobacteriaceae* and *Vibrionaceae* (Carraro et al., 2016; Partridge et al., 2018).

#### Integrative Mobilizable Elements/Mobilizable Transposons

Similar to ICEs, the IMEs, also known as mobilizable transposons, are mobile elements that lack the recombination module that allows for their autonomous integration and excision into host sequence (Guedon et al., 2017; Lao et al., 2020). Unlike ICEs, which are self-transferable, IMEs cannot self-transfer. Instead they can be mobilized *in trans* by subverting the conjugation machinery of related or unrelated co-resident conjugative element to promote their own transfer (Guedon et al., 2017; Lao et al., 2020). Beside AMR genes, IMEs can carry genes conferring various known or unknown functions that may enhance the fitness of their hosts and contribute to their maintenance in bacterial populations (Doublet et al., 2005; Guedon et al., 2017). An example is SGI1, which was classified within the group of IMEs since it is not self-transmissible, but mobilizable (Doublet et al., 2005; de Curraize et al., 2021). SGI1 could be conjugally transferred from an *S. enterica* donor to *E. coli* recipient strains (Doublet et al., 2005). SGI1 has been shown to exploit the conjugal apparatus of the IncA/C plasmids to facilitate SGI1 being conjugally mobilized (Douard et al., 2010; Harmer et al., 2016; Siebor et al., 2016). SGI1 is extremely stable in the host chromosome; however, the presence of an IncA/C plasmid enables excision of SGI1 *via* the action of the IncC transcriptional activator complex AcaCD. This complex is required for expression of *tra* genes and hence for conjugative activity of IncC plasmids (Harmer et al., 2016; Huguet et al., 2016). AcaCD binds to SGI1 upstream of *xis*, which encodes the recombination directionality factor Xis, resulting in the activation of *xis* expression and hence SGI1 excision which enables subsequent mobilization of SGI1 (Kiss et al., 2015). The extra-chromosomal circle of SGI1 is integrated into the recipient chromosome in a site-specific manner (Doublet et al., 2005). Paradoxically, although SGI1 needs a co-resident IncA/C plasmid for its excision from the host chromosome and transfer to a new host, it is incompatible with A/C plasmids (Harmer et al., 2016; Huguet et al., 2016, 2020). SGI1 promotes the loss of IncA/C plasmid, which is associated with SGI1 replication. The high-copy replication of SGI1 prevents the co-transfer of IncA/C plasmid into a recipient cell (Harmer et al., 2016; Huguet et al., 2016, 2020). The mobilization of SGI1 from *Salmonella* to the other bacterial pathogens probably contributes to the spread of AMR genes among them.

### Insertion Sequences

In general, IS elements are small mobile elements (at least 0.5 kb) that perform as a complex type of transposable element and typically carry one or two transposase (Tnp) genes (Pagano et al., 2016; Partridge et al., 2018). These elements are defined as the simplest autonomous mobile elements in bacterial genomes (He et al., 2015). They are classified into different groups based on active site motifs in Tnp, designated by the key amino acids which are present in the active site including DDE (Asp and Glu), DEDO and H4H (a His split by a large collection of hydrophobic amino acids); and/or on whether transposition is a conservative, cut-and-paste mechanism, where the IS is simply excised from the donor and inserted into the recipient, or is replicative (Seth-Smith et al., 2012). The transposition can be replicated by a copy-and-paste mechanism or copy-out-paste-in mechanism. In the copy-and-paste mechanism, the IS is replicated to join the donor and recipient in a co-integrate, which is subsequently resolved to provide the original donor plus the recipient with the IS; while in the copy-out-paste-in mechanism, the IS is replicated into a double-stranded circular intermediate that then integrates into the recipient (Partridge et al., 2018). IS elements are widespread and can occur in very high numbers in bacterial genomes and have had an important impact on genome structure and function (Siguier et al., 2014). IS-mediated gene inactivation can affect bacterial virulence, AMR and metabolism (Vandecraen et al., 2017). For example, IS1 or IS10 insertion can up-regulate the AcrAB-TolC efflux pump in *Salmonella*, resulting in increased AMR (Ramirez-Santos et al., 1992; Siguier et al., 2014). IS1, which is approximately 768 bp, can be detected in the majority of the family *Enterobacteriaceae* including *E. coli*, *Salmonella*, *Shigella*, and *Klebsiella* (Ramirez-Santos et al., 1992). IS1 can be found in the chromosome and plasmids, respectively, and some strains carried IS1 in both the chromosome and plasmids (Ramirez-Santos et al., 1992). Ramirez-Santos et al. (1992) reported that the frequency of IS1 was higher in *Salmonella* than in *E. coli* and *Shigella*.

The relatively high prevalence of IS1 in plasmids of MDR clinical isolates suggests its role in the dissemination of AMR genes (Ramirez-Santos et al., 1992). ISPa12, a member of IS4 family, can induce expression of ESBL PER-1 in a series of Gram-negative isolates, including *Salmonella*, resulting in resistance to penicillins, cefotaxime, ceftibuten, ceftazidime, and aztreonam (Poirel et al., 2005). IS200 is a *Salmonella*-specific IS that is found in almost all *Salmonella* serotypes examined, but is absent from most other enteric bacteria, thus it has been used as fingerprinting tool that is applied for strain discrimination (Beuzon et al., 2004). IS elements have been reviewed thoroughly in previous publications (Siguier et al., 2014; Vandecraen et al., 2017).

### Resistance Islands/*Salmonella* Genomic Island

Resistance islands are genomic islands that contain multiple resistance genes. They have been described mainly in the Proteobacteria, including *Salmonella*, *Shigella flexneri*, *V. cholerae*, *Staphylococcus aureus*, and *Acinetobacter baumannii* (Pagano et al., 2016). Several AMR islands, also called SGIs, have been identified in *Salmonella* strains (Miriagou et al., 2006; Silva et al., 2012). The best known is SGI1, which was initially described in the epidemic MDR *Salmonella* Typhimurium phage-type DT104 (Boyd et al., 2001; Doublet et al., 2005). Since then, several variants of SGI1 have been described in a wide variety of *Salmonella* serovars, including Typhimurium, Agona, Paratyphi B, Albany, Meleagridis, and Newport, which indicates its horizontal transfer potential (Levings et al., 2005). SGI1 is 43 kb and contains 44 open reading frames (ORFs), many of which show no homology to known gene sequences. As mentioned above, SGI1 is an integrative mobilizable element that contains a complex class 1 integron named In104, located within the AMR gene cluster which is 13 kb and is located at the 3′ end of SGI1 (Doublet et al., 2005; Levings et al., 2005). The SGI1 antimicrobial resistance gene cluster contains a complex class 1 integron that harbors five resistance genes [*aadA2*, *sul1*, *floR*, *tetA(G)*, *blaPSE-1*] encoding ampicillin, chloramphenicol, florfenicol, streptomycin/spectinomycin, sulphonamides, and tetracycline (ACSSuT) resistance, the so-called ACSSuT resistance type (Doublet et al., 2005; Levings et al., 2005; de Curraize et al., 2021).

## Factors That Impact Resistance Transfer

Environmental and genetic factors that regulate horizontal transfer of AMR genes in bacterial populations remain mostly unknown. MDR *Salmonella* often have multiple resistance-encoding plasmids. The exposure to some antimicrobial agents appears to influence plasmid transfer in enteric bacteria under certain circumstances (Schuurmans et al., 2014; Moller et al., 2017; Lu et al., 2018; Shun-Mei et al., 2018; Liu et al., 2019). Several factors have been shown to contribute to the dissemination of resistance plasmids and other MGEs. Quorum-sensing (QS) and SOS responses have been known to promote horizontal gene transfer, which plays an essential role for the AMR development and dissemination among bacterial populations (Lu et al., 2017; Shun-Mei et al., 2018; Liu et al., 2019).

### Quorum-Sensing System

Quorum-sensing is an intercellular cell–cell communication mechanism that allows bacteria to control the expression of genes involved in a variety of cellular processes and plays a critical role in the adaption and survival of bacteria in their environment (Kendall and Sperandio, 2007). QS is mediated by secreted chemical signals called autoinducers (AIs), which are small diffusible molecules that are synthesized and released from bacterial cells in accordance with cell number and accumulate in the external environment (Taga et al., 2003). When bacterial cell density reaches to a certain level that AIs concentration is over a minimal threshold, AIs bind to cognate receptors to alter and regulate bacterial gene expression accordingly in response to their population density (Taga et al., 2003; Kendall and Sperandio, 2007). A large number of physiological processes in bacteria, including biofilm formation, virulence factor production, bioluminescence, sporulation, motility, and antibiotic production, are regulated by QS systems (Rutherford and Bassler, 2012; Wang et al., 2019a).

While most QS signals are species-specific, AI-2 has been observed throughout the bacterial kingdom and is considered as a universal QS signal (Wang et al., 2019a). It is involved in interspecies cell-to-cell communication, as AI-2 produced by one species can influence gene expression in another. AI-2 is a metabolic byproduct of a *luxS* gene-encoded synthase, which is an enzyme that has been found in more than 55 bacterial species and is involved primarily in the conversion of ribosyl-homocysteine into homocysteine and 4,5-dihydroxy-2,3-pentanedione (DPD), the precursor of AI-2 (Vendeville et al., 2005). The LuxS/AI-2 QS system is biologically important as it is involved in numerous physiological processes such as altering bacterial growth characteristics, biofilm formation, conjugation, virulence regulation, AMR, and metabolism in *Salmonella* (Taga et al., 2001; Choi et al., 2007; Widmer et al., 2007; Chen et al., 2008; Karavolos et al., 2008; Jesudhasan et al., 2010; Ju et al., 2018; Shi et al., 2018). It contributes to AMR through different mechanisms: MGE, efflux pumps, the VraSR two-component system, inhibition of the folate synthesis pathway, and biofilm formation. Detailed information on the relationships between the LuxS/AI-2 system and drug resistance has been reviewed by Wang et al. (2019a). In brief, the LuxS/AI-2 system has been shown to impact the transfer of resistance plasmids and the LuxS/AI-2-based QS enhances the expression of genes involved in conjugation-related activities, leading to increased conjugation frequency (Lu, 2004).

In the presence of AI-2, the conjugation frequency of the plasmid RP4 carrying the *tet(A)* gene in bacteria exposed to tetracycline increased significantly compared to a non-exposed control (Lu, 2004). Increased AI-2 concentrations led to increased expression of plasmid-transfer associated gene *trbC*. Similar studies conducted by another group also showed that exposure to sub-MIC tetracyclines could facilitate the conjugative transfer of plasmid RP4 in *E. coli* and this process could be enhanced by AIs, but inhibited by quorum sensing inhibitors (QSIs; Zhang et al., 2018). Therefore, the LuxS/AI-2 could be a potential target for preventing the spread of bacterial resistance.

### SOS Response

**Figure 4:**
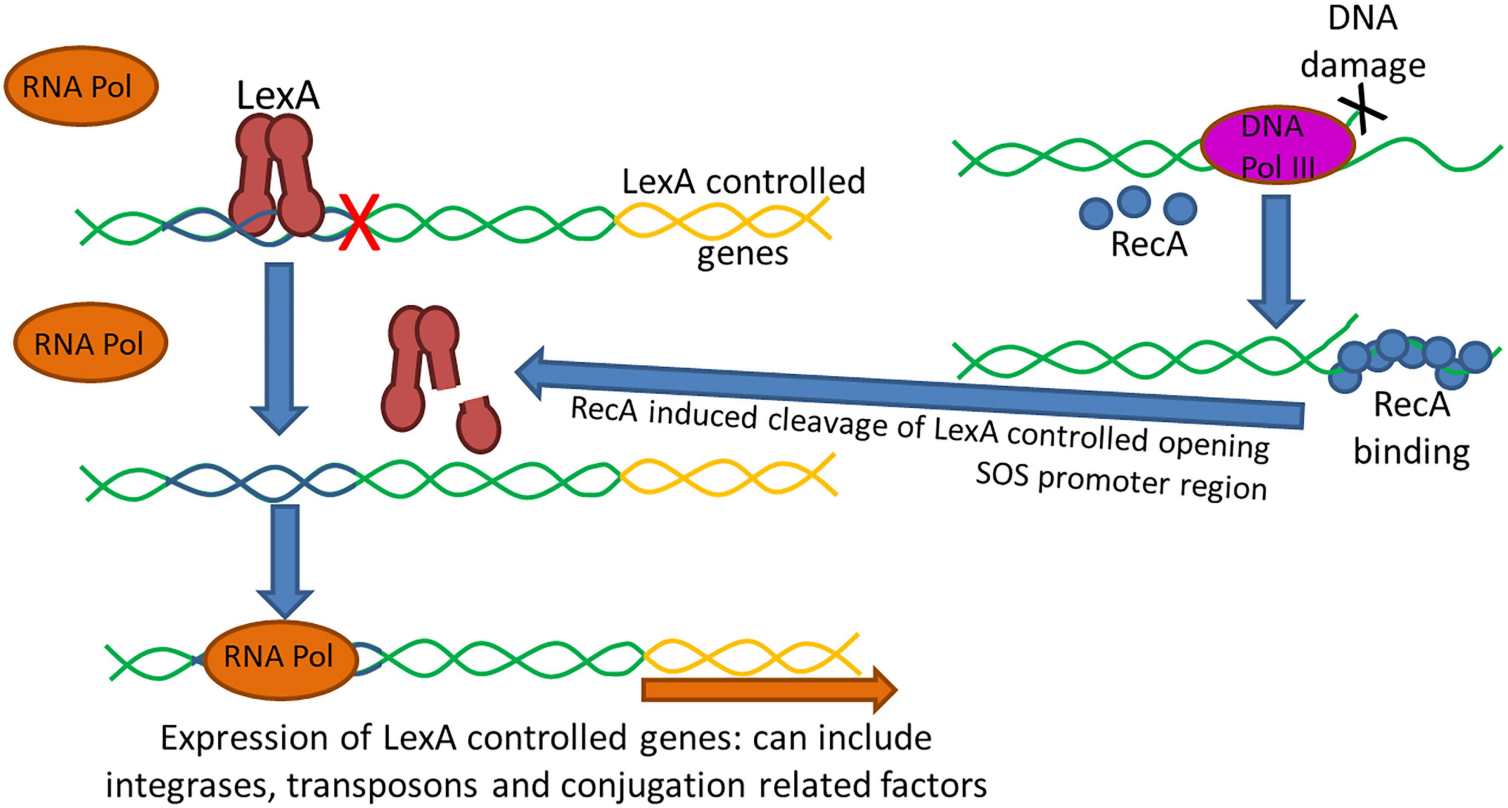
The SOS response is an inducible DNA repair pathway controlled by two key regulators, the LexA repressor of gene expression and the RecA inducer. Under normal conditions, LexA is bound to the promoter of SOS-associated genes preventing binding of RNA polymerase (RNA Pol) and expression of the regulated genes (Maslowska et al., 2019). Following DNA damage, such as following certain antimicrobial exposures, the DNA polymerase III (DNA Pol III) enzyme senses the damage and the increase in single-stranded DNA (ssDNA) causes RecA to become activated and bind to the ssDNA. The ssDNA/RecA complex leads to proteolysis of the LexA repressor allowing RNA polymerase to bind facilitating expression of genes under LexA control. LexA has been shown to impact the expression of integrases and transposases, as such the activation of the SOS response has been shown to promote horizontal dissemination of resistance genes. Additionally, conjugation can also trigger the SOS response as ssDNA is generated and transferred during the conjugation process which can upregulate integrase and transposase expression and triggering recombination of plasmids in the recipient cells.

The SOS response is a global stress response to DNA damage in which the cell cycle is arrested and DNA repair and mutagenesis is induced (Beaber et al., 2004; Baharoglu et al., 2010; Zgur-Bertok, 2013; Qin et al., 2015). It is an inducible DNA repair pathway controlled by two key regulators, LexA, a repressor, and RecA, an inducer (Podlesek and Žgur Bertok, 2020; Figure 4). In bacteria, upon DNA damage, SOS is induced when an increase in intracellular single-stranded DNA (ssDNA) is generated when DNA polymerase III stalls at a lesion while helicase continues unwinding DNA (Podlesek and Žgur Bertok, 2020). Stimulated by ssDNA, RecA is activated by binding to ssDNA (Maslowska et al., 2019). The formation of a ssDNA/RecA nucleofilament stimulates auto-proteolysis of the LexA repressor, leading to de-repression of genes comprising the SOS regulon (Baharoglu et al., 2010; Podlesek and Žgur Bertok, 2020). In addition to several endogenous triggers (for example, UV irradiation, oxidants, and chemical mutagens) that cause the accumulation of ssDNA, thus inducing the SOS response, ssDNA is also produced by several mechanisms of exogenous DNA uptake involved in lateral gene transfer, including conjugation, transformation, and occasionally transduction (Baharoglu et al., 2010). The SOS response can also be induced by numerous antimicrobials, presumably because these antimicrobials cause the production of ssDNA. At least two classes of antimicrobials, fluoroquinolones (such as ciprofloxacin) and dihydrofolate reductase inhibitors (such as trimethoprim), can activate the SOS response (Beaber et al., 2004; Qin et al., 2015). Besides being initially recognized as a regulator of DNA damage repair, it has been noted that the SOS response plays a much broader role in bacteria. As an error-prone repair system, the SOS response contributes significantly to DNA changes observed in a wide range of species by promoting an elevated mutation rate which generates genetic diversity and adaptation, including the development of resistance to antimicrobials (Podlesek and Žgur Bertok, 2020). The SOS response also plays a key role in AMR by stimulating gene transfer, inducing mutation and genomic rearrangements, and the formation of biofilms which are highly recalcitrant to antimicrobials (Podlesek and Žgur Bertok, 2020).

The SOS response has been shown to promote horizontal dissemination of resistance genes in the presence of antimicrobial agents (Beaber et al., 2004; Hastings et al., 2004). Guerin et al. (2009) observed that the antimicrobial compounds found in manure can activate the SOS response in bacteria, which then increases the activity of integrases and transposases, and thereby transposition rates of gene cassettes and other translocative elements (Guerin et al., 2009). They concluded that LexA controlled expression of most integron integrases and consequently regulated cassette recombination. Baharoglu et al. (2010) found that conjugative transfer of plasmids R388, R6Kdrd, and RP4 in *E. coli* and *V. cholerae* induces the SOS response and up-regulates integrase expression, triggering cassette recombination in recipient cells (Baharoglu et al., 2010). Additional studies have shown that the SOS response can lead to an increase in cell membrane permeability and downregulation of the *korA* repressor of various transfer associated genes in the RP4 plasmid (Wang et al., 2019b).

The SOS induction leads to genetic diversification of these mobile elements and their transfer to surrounding bacteria, resulting in the dissemination of AMR genes (Baharoglu et al., 2010). It was also observed that ICEs could utilize the SOS response to mobilize themselves from the bacterial chromosome and infect other cells (Hastings et al., 2004). The transfer of SXT, an approximately 100 kb ICE derived from *V. cholerae* that encodes a variety of antimicrobial resistance genes, is markedly enhanced when the SOS response is induced by two DNA-damaging antimicrobials (mitomycin C and ciprofloxacin), resulting in the transfer of MDR genes (Hastings et al., 2004).

Other studies have outlined the connections between horizontal gene transfer, bacterial stress response (SOS), and recombination of gene cassettes in integrons, which provides new insights into the development of the AMR within a population (Guerin et al., 2009; Baharoglu et al., 2010). Since several commonly used antimicrobials can induce the SOS response, which in turn can enhance the conjugative transfer of the plasmids, the use of certain antimicrobial agents, either clinically or in agricultural settings, might potentiate the horizontal dissemination of AMR genes to a broad range of bacterial species and hasten genetic change and the evolution to resistance in pathogenic populations.

## Conclusion

Antimicrobial resistance acquisition can occur *via* either vertical or horizontal transfer. In vertical transmission, the accumulated genetic errors in existing genes (either in the chromosome or plasmid) is passed on from parent cells to progeny cells, leading to the observed resistance. While in the horizotal transfer, also called as acquired resistance, the AMR genes are exchanged within and among different bacteria species (Founou et al., 2016). The resistant genes harbored on MGEs can be acquired by the recipient strains. Both vertical transmission and horizontal transfer of AMR genes play important roles in AMR acquisition. The MGE-mediated horizontal transfer of foreign genes among different bacteria plays an important role in bacterial evolution and contributes signaficatnly in both the AMR and virulence gene acquisition (Gyles and Boerlin, 2014). This review article examined MGEs that can be found in *Salmonella* as well as discussed their roles in the development and dissemination of antimicrobial resistance. Key examples of MGEs that are important in antimicrobial resistance dissemination include SG1, which has been associated with the stable acquisition of multiple resistance genes at a site in the chromosome, yet requires an IncC resistance plasmid to facilitate its transfer. Plasmids are likely the most important vehicle of resistance transfer between different *Salmonella* strains and/or from related species. Plasmids can harbor other MGEs including transposons and integrons that can lead to the transfer of genes between the chromosome and other co-resident plasmids, thus providing more genetic diversity among the bacterial populations. The dynamic resistance of *Salmonella* isolates to antimicrobial agents is considered a serious problem and developing methods and strategies to reduce and control the spread of resistance genes and their evolution are vital. Another trend observed in *S. enterica* is IS element activation which plays a critical role in efflux mechanisms. A recent study suggested that a combination of conjugation inhibition and plasmid elimination would be considered an effective method to reduce the conjugation-assisted persistence of antimicrobial resistance (Lopatkin et al., 2017; San Millan, 2018). Since the induction of SOS response promotes horizontal dissemination of AMR genes, inhibiting the bacterial SOS response would be a suitable target to prevent the acquisition of AMR genes, and could be used in combination with antibiotics for the treatment of infections (Baharoglu et al., 2010). The different types MGEs are summarized in Table 4, which describes the antimicrobial resistance mobilome of zoonotic pathogens, particularly in *S. enterica*, that is critical to understand the epidemiology, dynamics and evolution of antimicrobial resistance.

**Table 4:** Summary of terms and definitions of mobile genetics elements (MGEs) in *Salmonella enterica*.

Mobile genetic elements	Definition
Plasmids	Plasmids are genetic elements that are typically circular DNA in a cell that can replicate independently of the chromosomes. They have variable host-ranges and can be conjugative and replicate in diverse genera.
Integrons	Integrons are genetic elements with a site-specific recombination system that can integrate, express and exchange specific DNA elements (gene cassettes), including resistance genes.
Transposable elements/Transposons (Tn)	Transposable elements/ Transposons (Tn) are known as “jumping genes” that can move themselves (and associated resistance genes) almost randomly to new locations in the same or different DNA molecules within a single cell.
Resistance islands	Resistance islands are genomic islands (GIs) containing MDR genes.
Bacteriophages	Bacteriophages are bacterial viruses that infect and replicate only in bacterial cells.
Insertion sequences	Insertion sequences are small mobile elements (generally 700 ~ 2,500 bp) and only code for proteins implicated in the transposition activity. They can move themselves almost randomly to new locations in the same or different DNA molecules within a single cell.

## Author Contributions

SA and JH contributed equally to this work and manuscript writing. SR and SF conceived the idea and assisted with the manuscript writing. All authors contributed to the article and approved the submitted version.

## Author Disclaimer

This manuscript reflects the views of the authors and does not necessarily reflect those of the United States Food and Drug Administration. Any mention of commercial products is for clarification only and is not intended as approval, endorsement, or recommendation.

## Conflict of Interest

The authors declare that the research was conducted in the absence of any commercial or financial relationships that could be construed as a potential conflict of interest.

## Publisher’s Note

All claims expressed in this article are solely those of the authors and do not necessarily represent those of their affiliated organizations, or those of the publisher, the editors and the reviewers. Any product that may be evaluated in this article, or claim that may be made by its manufacturer, is not guaranteed or endorsed by the publisher.
